# Supplementary material for: Benzodiazepine Boom: Tracking Etizolam, Pyrazolam, and Flubromazepam from Pre-UK Psychoactive Act 2016 to Present Using Analytical and Social Listening Techniques
Source: Pharmacy (Basel). 2024 Jan 12;12(1):13. doi: 10.3390/pharmacy12010013 (PMC10801481; doi:10.3390/pharmacy12010013)
Supplement: Supplementary file 1 [file pharmacy-12-00013-s001.zip › pharmacy-2713961-supplementary.pdf]

Supplementary Materials

Appendix S1:

Reference and Sample Preparation

UHPLC-MS Analysis: *Internal Standard (WSIS):* A single diazepam working stock solution at 0.08 mg/mL (80 µg/mL) was created by diluting 40 mg Diazepam reference standard to 500mL with methanol, to provide a WSIS; the resulting mixture was vortexed (Level 4, Vortex Genie 2, Scientific Industries, N.Y., USA) for 2 min, and sonicated for 5 minutes (25°C) to complete the API extraction. The resulting supernatant was finally filtered through 13 mm 0.22 µm PTFE syringe filter [Thermo Fisher Scientific]. *Reference Standards:* 3mg of each drug reference standard diluted to 10mL in methanol, gave 10mL solution at 0.3mg/mL (300µg/mL). Each standard was prepared in triplicate for each drug (RSWS1, RSWS2, RSWS3); the resulting mixture was vortexed (Level 4, Vortex Genie 2, Scientific Industries, N.Y., USA) for 2 min, and sonicated for 5 min (25°C) to complete the API extraction. The resulting supernatant was finally filtered through 13 mm 0.22 µm PTFE syringe filter (Thermo Fisher Scientific). Additional dilutions were carried out from each RSWS and WSIS, to offer a calibration range of 6 reference standard concentration points between 0.0075mg/mL (7.5 µg/mL) and 0.09 mg /mL (90 µg/mL), with a fixed IS concentration at 0.04 mg /mL (4 µg /mL). To each calibration dilution, a fixed volume of WSIS was added to provide an Internal Standard Addition at 0.04 mg/mL (40 µg/mL). *Sample Preparation:* Etizolam and Pyrazolam: [Step 1] Samples prepared in triplicate working stock solutions (SWS1, SWS2, SWS3) by crushing each sample in a pharmaceutical pestle and mortar and accurately weighing each tablet pre and post maceration. Crushed materials were dissolved to 10mL in methanol; the resulting mixture was vortexed (Level 4, Vortex Genie 2, Scientific Industries, N.Y., USA) for 2 minutes, and sonicated for 5 minutes (25°C) to complete the API extraction. The resulting supernatant was finally filtered through 13 mm 0.22 µm PTFE syringe filter [Thermo Fisher Scientific], and 8 mL of the supernatant was re-diluted to 10 mL in a grade A volumetric flask. [Step 2] an 8mL aliquot of {Step 1] was further diluted to 10 mL in methanol to provide an estimated sample concentration of 0.08 mg /mL (80 µg /mL). The proposed sample concentration of Flubromazepam was much higher, at ~8 mg API per tablet, therefore a 1mL aliquot of {Step 1} solution was re-diluted to 10 mL to provide a proposed sample concentration for test of 0.08 mg/mL (80 µg /mL). Equal volumes of RSWS/WSIS and SWS were extracted using a glass pipette and added to a grade a volumetric flask to provide reference calibration, a fixed internal standard and sample for robust qualitative sample analysis. A single 1mL aliquot of each prepared sample was extracted directly into a Waters 12x32 mm UHPLC-MS glass chromatography vial [Waters UK] ready for analysis.

An internal standard addition method was employed, using a diazepam reference standard, MeOH diluted to give 0.04mg /mL (40µg/mL), per prepared sample. This was deemed advantageous as it exhibits a proportional response to variations in the analyte, yielding a similar yet not identical measurement signal; its absence from the sample matrix ensures the exclusive presence of the internal standard, enhancing accuracy and reliability in the analysis.

NMR Analysis. *NMR Reference Samples:* ~1mg of each drug reference standard diluted to 1mL deuterated methanol (MeOH-d4), vortexed (Level 4, Vortex Genie 2, Scientific Industries, N.Y., USA) for 2 min, and sonicated for 5 min (25°C) to complete the API extraction. The

resulting supernatant was finally filtered through 13 mm diameter PTFE syringe filter, with a 0.22 μm pore size (Thermo Fisher Scientific) and individually transferred to nitrogen cleaned Wilmad 5 mm Thin Wall Precision NMR Sample Tube for analysis. *Street Sample NMR:* 3mg of material from each tablet was dissolved in 1.2 mL of MeOH-d4, vortexed (Level 4, Vortex Genie 2, Scientific Industries, N.Y., USA) for 2 min, and sonicated for 5 min (25°C) to complete the API extraction. The resulting supernatant was finally filtered through 13 mm diameter PTFE syringe filter, with a 0.22 μm pore size (Thermo Fisher Scientific) and individually transferred to nitrogen cleaned Wilmad 5 mm Thin Wall Precision NMR Sample Tube for analysis.

Appendix S2:

Results

UHPLC-MS(MS)

1. **Etizolam Reference Standard and ET1-6 Tablets**

Structural Analysis. UHPLC-MS(MS) analysis of the analytical grade etizolam reference standard (Figure 2A) resulted in a single chromophore response at 2.45 min, with the internal standard addition, diazepam, eluting at 2.73 min (Table 2A). The same 2 chromophore responses were visible within the tablet samples (ET1-ET6 (B1-3)), within the expected peak elution timeframes. Both lone peaks were compared with literature (Cayman Chemicals, 2023) to confirm the chromophore presence expected from the etizolam molecule. The ESI spectra for all samples showed molecular ions at m/z 343 and 345, along with a visible 3:1 ratio between the reported ions, therefore indicating $^{35}Cl/^{37}Cl$ isotopes related to the chlorine atom attached to the thiophene ring, associated with the etizolam chemical structure. The raised molecular ion mass at 345 is evidence of an MH^+ peak, a by-product of the UHPLC-MS(MS) process, when coupled with an expected 3:1 ratio within MS, further supporting the presence of the chlorine atom. The diode array detector (DAD) response could not confirm the presence of a single compound as etizolam and diazepam maximum absorbance (λmax) for the two compounds coincide, at 251 nm and 245 nm respectively. With adjustments for the MH^+, the compound of interest in all tablet samples is likely to be a mass of ca. 342.8 g/mol^{-1}, concurring with literature.

Tablet Purity Analysis. Comparison of etizolam tablets shows high inter-batch and sample purity variability. For example, etizolam tablet sample 5, analysed in triple-triplicate batches 1-3 (ET5 B1-3), showed the lowest concentration of all tablet samples in batch 2, with an average drug content of 13.8 ± 0.6 mg. Sample ET1 B2 showed the highest purity at 24.7 ± 0.9 mg. Whilst samples ET5 B1-3 resulted in the lowest concentration range of all etizolam batches, ranging from 13.8 ± 0.6 mg and 15.5 ± 0.6 mg; the highest concentration cluster of sample batches was found within sample ET4 B1-3, ranging from 23.6 ± 1.4 mg to 24.7 ± 0.8 mg. At the point of purchase the suggested purity for each batch of tablets was proposed as 1 mg of the etizolam API; as a minimum, these tested samples show a maximum increase in purity of 2450% increase in purity, compared with the proposed drug content (Tables 2A).

2. **Flubromazepam Reference Standard and FT1-5 Tablets**

Structural Analysis. UHPLC-MS(MS) analysis of the flubromazepam reference material (Figure 2B) resulted shows a single chromophore response at 2.45 min, with the internal standard addition, diazepam, eluting at 2.68 min (Table 2B). Both peak elution profiles were visible across every tablet sample (FT1-FT5(B1-3)), concordant with the reference standard spectra. Both lone peaks were compared with literature (Cayman Chemicals, 2023) to confirm the chromophore response was concurrent with that expected of the flubromazepam molecule. ESI spectra for all samples showed protonated molecular ions at m/z 333 and 335 (Table 2B); corresponding with the average molecular mass at 333.15 g/moL^{-1}. The DAD response showed a λmax around 268 nm, typically within the region expected for a BZD derivative; with some overlap expected from the diazepam compound.

Tablet Purity Analysis. Comparison of flubromazepam tablets shows a high inter-batch and sample purity variability. Flubromazepam tablet sample 1, analysed in triple-triplicate batches 1-3 (FT1 B1-3), showed the lowest concentration of all tablet samples in batch 2, at 4.0±0.2 mg. Sample FT5 B2 resulted in the highest purity, 23.5±0.8 mg. Samples FT1 B1-3 resulted in the lowest concentration range of all flubromazepam batches, ranging from 4.0±0.2 mg and 4.5±0.2mg, and the highest concentration cluster of samples was found within sample FT5 B1-3, ranging from 22.7±0.7mg to 23.5±0.8 mg. The largest inter-batch variation is found within FT2 B1-3, varying between, 14.2±0.6 mg and 22.2±0.7 mg. At the point of purchase the suggested purity for each batch of tablets was proposed as 8 mg of the flubromazepam API; as a minimum, these tested samples show a maximum increase in purity of 194%, compared with the advertised drug content (Table 3).

3. **Pyrazolam Reference Standard and PT1-4 Tablets**

Structural Analysis. Interrogation of the pyrazolam reference material (Fig 2C) shows a single chromophore response for pyrazolam at 1.02 min, with the internal standard addition, diazepam, eluting at 2.68 min (Table 2C). Peak elution profiles were visible across every tablet sample (FT1-FT5), as within the reference standard spectra. Each lone peak was compared with literature (Cayman Chemicals, 2023) to confirm the chromophore response as expected from a pyrazolam molecule. ESI spectra for all samples showed molecular ions at m/z 354 and 356. Pyrazolam, with a molecular mass of 354.21 g/moL^{-1}, the parent ion and MS fragmentation patterns support the presence of pyrazolam within this tablet sample. The reported DAD response showed a λmax around 254 nm, typically within the region expected for a benzodiazepine derivative; there was evidence of UV overlap from the diazepam compound. No evidence was found to suggest the presence of an MH^+ peak which is concordant with the pyrazolam structure. Peak signal from each tablet is much weaker than with the internal reference standard, a weak signal of this magnitude may indicate the concentration of pyrazolam within the tablet samples is very low, compared to the known concentration of the reference standard.

Tablet Purity Analysis. Comparison of pyrazolam tablet samples indicate a high inter-batch and sample purity variability. Pyrazolam tablet sample 1, analysed in triple-triplicate batches 1-3 (FT1 B1-3), showed the lowest concentration of all tablet samples in batch 1, at 5.4±0.2 mg. Sample FT4 B2 resulted in the highest purity, 11.5±0.4 mg. Samples FT1 B1-3 resulted in the lowest concentration range of all pyrazolam batches, ranging from 5.4±0.2 mg and 8.2±0.3 mg, and the highest concentration range within pyrazolam batches identified within samples FT4 B1-3, ranging from 10.5±0.3 mg to 11.5±0.4 mg. The largest inter-batch variation is found within PT2 B1-3, varying between, 7.1±0.3 mg and 11.1±0.4 mg. At the point of purchase the suggested purity for each batch of tablets was proposed as 1 mg of the pyrazolam API; as a minimum, across all tested samples and batches, this represents a maximum increase in purity of 1050% compared with the stated drug content (Table 3).

1H NMR Interrogation

Confirmation of the appropriate benzodiazepine was considered achieved if all the peaks seen in the reference sample were also seen in the drug sample with the correct number of protons per peak. After integrating drug sample spectrum any peaks with a large integral value (> 10)

were considered to be excipients and ignored for comparison purposes. NMR as a confirmatory technique, confirmed the structures within all reference standards, however, appropriate drug content could only be identified in flubromazepam and pyrazolam tablet batches.

1. **Etizolam**

As expected, four protons from the aromatic ring were seen around 7.5 ppm, a single proton at 6.5 ppm from the thiophene ring together with a two proton quartet at 2.85 ppm (from ethyl side chain), a three proton singlet at 2.7 ppm from methyl group on azole ring and a three proton triplet at 1.3 ppm from the ethyl side chain (Table 2A). As mentioned above, no peaks from the methylene protons of the seven membered ring could be seen. Across all drug samples, the methyl proton signal expected from the ethyl side chain could not be identified; although this may be a result of excipients obscuring the signal in the aliphatic region, around 1.3 ppm. Sample ET1, ET2 and ET3 showed peaks at 4.48 ppm and 4.34 ppm, which may possibly indicate methylene protons expected from the seven membered ring. However, sample ET4, ET5 and ET6 did not show these peaks. Sample ET5 showed a weak noisy spectrum with only the aromatic protons being detected whereas ET6 showed a spectrum similar to ET4. Table 3 indicates ET5 did have a lower average drug content than the other samples. Hence, it was not possible to confirm the presence of etizolam in any of the tested drug samples, by ^{1}H NMR alone. Theoretical assessment of the etizolam chemical structure suggests that two methylene proton peaks should be expected, associated with the seven membered ring. The SWG database shows a single broad peak at 4.9 ppm containing two protons; however, this was run in deuterated DMSO (SWGDrug, 2023). The standards and samples in this study were run in deuterated methanol, so possibly the methylene protons are swamped by the water signal from the methanol which is expected at around 4.8 ppm.

2. Flubromazepam

All drug samples showed the same peaks as evident in the analysed flubromazepam reference standard (Table 2A), confirming the presence of flubromazepam in the drug samples, by proton NMR, has been achieved. It should be noted however that we did not see the NH proton signal in the reference material which ought to be seen around 10 ppm. This may be due to exchange of the amino proton with deuterium from deuterated methanol. However, all other peaks have been well documented in (Ligon *et al*, 2019), enabling the confidence to declare the flubromazepam compound within the reference material and all samples FT1-FT5.

2. **Pyrazolam**

All drug samples showed the pyrazolam peaks seen in the reference pyrazolam proton spectrum (Table 2A), more strongly in PT2 and PT4 than in PT1 and PT3. Compared with the calculated purity levels (Table 3), it is evident that samples PT2 and PT4 contain an increased drug content, compared to PT1 and PT3; supporting the theory that these sample would contain a higher signal under NMR. Therefore, the presence of pyrazolam in the reference material and all pyrazolam drug samples was confirmed by proton NMR with confidence.

Appendix S3:

Examples of manual thematic analysis of data collected from social media listening

Manual Thematic Analysis	
**Ranked Analysis of Social Media Data**	**Relevant Social Media Posts**
**Harms** – Was identified as the most prevalent theme. Included within this group are posts expressing concerns about dosing, potential side-effects, withdrawals, and the risks associated with using these substances. Also grouped into this are discussions around safety concerns and health effects, which include discussions about tapering off substances and avoiding withdrawals. We also recognise several concerns about counterfeit substances and risks associated with purchasing from unverified sources.	- I went into kidney failure and had to be Narcan three times after using 3HOPCP and etizolam<br>- Need Help About Etizolam Dependance/When to tapper and Advice about my situation<br>- im super sickkk just took a roxy with 2mg clonazapam and half of bar of etizolam<br>- A side effect of continual pyrazolam use was double vision, it's such a strong benzo with weak effect<br>- Pyrazolam extreme overdose<br>- I'm [redacted] terrified tapering off of Flubromazepam using Valium, I'm stuck at home in isolation and don't have the motivation to do anything, I don't enjoy anything anymore Feelings of Self-harm or Suicide
**Buy / Sell** - The second most prevalent theme involves members of the community of PWUD seeking or offering advice about the availability of these specific DBZDs, even when termed as research chemicals. Here we record an emphasis on sourcing drugs through open channels. We also recognise several concerns about risks associated with purchasing from unverified sources.	- Hot selling etizolam alprazolam bromazolam flubromazepam flubrotizolam pyrazolam 90212-80-9 40054-69-1 28981-97-7 71368-80-4 2647-50-9 WhatsApp [redacted]<br>- etizolam alprazolam bromazolam flubromazepam flubrotizolam pyrazolam 90212-80-9 40054-69-1 28981-97-7 71368-80-4 2647-50-9 wickr me: [redacted]<br>- Pyrazolam Pellets 3mg Effects Wholesale EU Online Supplier<br>- BRING BACK THE ETIZOLAMS AND PYRAZOLAMS, BRING THEM BACK!
**Effects** – The third most prevalent topic manually identified is that of positive and negative effects from these three DBZDs, including their effects, specific usage patterns, and poly-substance patterns. Users share their experiences, positive outcomes, and concerns about side effects.	- Trouble sleeping after taking lower doses of pyrazolam<br>Flubromazepam. Beautiful anxiolytic effects, very long half-life, outstanding for GAD and other anxiety disorders<br>- Flubromazepam is a very long acting for a benzodiazepine derivative - three whole days.
**Other** – The fourth most common theme was an open theme, where discussions around education, seizures, GO/NGO statements regarding matters surrounding these 3 substances.	- Two new monographs were added today (Isobutyryl Fentanyl and Flubromazepam)<br>- Vice News is sensationalizing Etizolam in their fentanyl reporting.<br>- Vice News is sensationalizing Etizolam in their fentanyl reporting.<br>Any reagents for testing benzo/etizolam? Discussion

**Trends –** The fifth most prevalent topic within the manual analysis is that detailing very specific usage patterns, and primarily new and/or reportedly effective poly-substance patterns. Users share their experiences, positive and negative outcomes, and also concerns.	- 1st thing i did when i woke up was pop a bar, 5mg of etizolam and 2 fat lines of oxy what am i doing<br>- bic, etizolam/clonazolam tablets/pure powder, adderall ir, suboxone, trazadone, seroquel, blu e-cig, straw, scissors<br>- Does anyone know something about Etizolam and Tianeptine sodium???<br>- Is it ok for me to combine pyrazolam and flubrotizolam?

Appendix S4

Etizolam ^{1}H NMR assignments

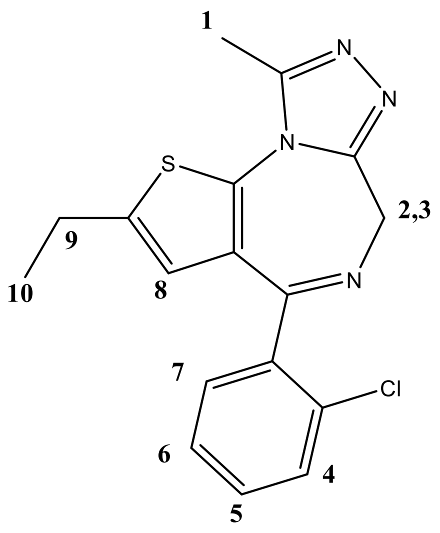

**Signal**	**Chemical Shift(s) ppm**	**Notes**
1	2.71 (s)	
2,3	not seen!	
4 – 7	7.52 – 7.42 (m, 4H)	
8	6.47 (s)	
9	2.82 (q, 2H 7.6 Hz)	
10	1.28 (t, 3H 7.6 Hz)	

Flubromazepam ^{1}H NMR assignments

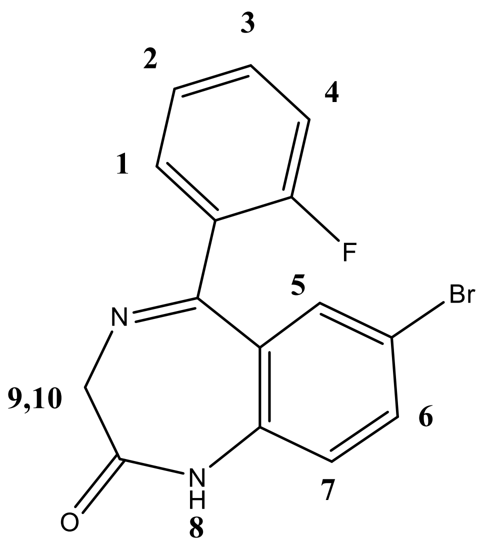

Signal	Peak	Notes
8	~ 10	not seen
4	7.68 (m)	most likely as close to electronegative F
5,6	7.56 (m, 2H)	close to Br atom
1,2,3,7	7.28 (m, 2H), 7.16 (m, 2H)	
9,10	4.28 (s broad, 2H)	methylene proton signals

Pyrazolam ^{1}H NMR assignments

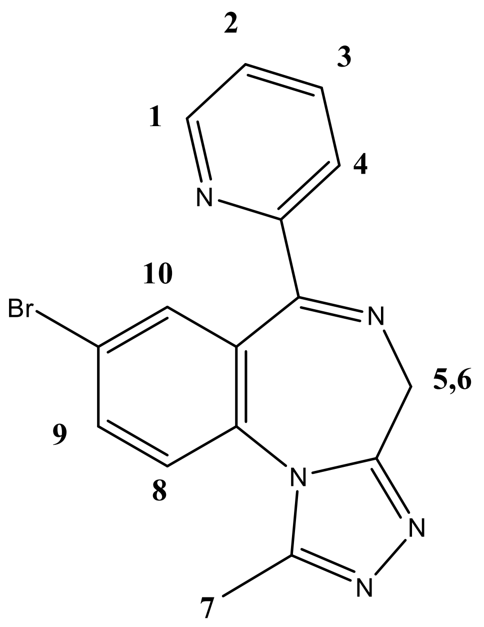

Signal	Peak	Notes
1	8.52 (d)	closest to N atom in ring, most deshielded
4, 9	8.07 (d), 7.69 (d)	
3	7.98 (m)	most likely as it does look like a doublet of doublets
2, 8, 10	7.93 (m), 7.52(m, 2H)	
5, 6	5.34 (d), 4.34 (d)	protons not free to rotate hence split each other
7	2.65 (s)	methyl protons on five membered ring side chain
